# Remembering Mechanosensitivity of NMDA Receptors

**DOI:** 10.3389/fncel.2019.00533

**Published:** 2019-12-05

**Authors:** Luke R. Johnson, Andrew R. Battle, Boris Martinac

**Affiliations:** ^1^Victor Chang Cardiac Research Institute, Darlinghurst, NSW, Australia; ^2^St. Vincent’s Clinical School, University of New South Wales, Darlinghurst, NSW, Australia; ^3^Division of Psychology, School of Medicine, University of Tasmania, Launceston, TAS, Australia; ^4^Department of Psychiatry, Center for the Study of Traumatic Stress, Uniformed Services University of the Health Sciences, Bethesda, MD, United States; ^5^School of Biomedical Sciences, Institute of Health and Biomedical Innovation (IHBI), Queensland University of Technology, Brisbane, QLD, Australia; ^6^Prince Charles Hospital Northside Clinical Unit, School of Clinical Medicine, The University of Queensland, Brisbane, QLD, Australia; ^7^Translational Research Institute, Woolloongabba, QLD, Australia

**Keywords:** memory, learning, NMDA, mechanobiology, amygdala, force, lipids

## Abstract

An increase in post-synaptic Ca^2+^ conductance through activation of the ionotropic N-methyl-D-aspartate receptor (NMDAR) and concomitant structural changes are essential for the initiation of long-term potentiation (LTP) and memory formation. Memories can be initiated by coincident events, as occurs in classical conditioning, where the NMDAR can act as a molecular coincidence detector. Binding of glutamate and glycine, together with depolarization of the postsynaptic cell membrane to remove the Mg^2+^ channel pore block, results in NMDAR opening for Ca^2+^ conductance. Accumulating evidence has implicated both force-from-lipids and protein tethering mechanisms for mechanosensory transduction in NMDAR, which has been demonstrated by both, membrane stretch and application of amphipathic molecules such as arachidonic acid (AA). The contribution of mechanosensitivity to memory formation and consolidation may be to increase activity of the NMDAR leading to facilitated memory formation. In this review we look back at the progress made toward understanding the physiological and pathological role of NMDA receptor channels in mechanobiology of the nervous system and consider these findings in like of their potential functional implications for memory formation. We examine recent studies identifying mechanisms of both NMDAR and other mechanosensitive channels and discuss functional implications including gain control of NMDA opening probability. Mechanobiology is a rapidly growing area of biology with many important implications for understanding form, function and pathology in the nervous system.

## Introduction

A key feature of the brain is its remarkable ability to form associations between coincident, or simultaneously linked events, allowing organisms to learn from the past and thereby predict the future. When individual neurons are coincidently active, they will promote a strengthening of synaptic connections, which forms the basis of the synaptic theory of associative memory (Langille and Brown, 2018). The later identification of associative long-term potentiation (LTP) has provided a cellular mechanism for Hebb’s first postulate on memory formation. This theory has proved tremendously informative, and extensive evidence (Johnson et al., 2008, 2009; Sah and Westbrook, 2008; Johansen et al., 2014) suggests that Hebb’s first postulate is a likely mechanism of learning and memory at the level of the individual neuron (Hebb, 1949). Since Hebb’s landmark theory, an extensive body of research has identified some of the key cellular and receptor mechanisms that underlie the formation of LTP. Memories are formed by plasticity at excitatory glutamatergic synapses in neural circuits, a process which depends on activation of ionotropic N-methyl-D-aspartate receptors (NMDAR). Activation of NMDAR is an essential step for memory acquisition leading to consolidation, which indicates that it is a crucial receptor for neural network activity and network plasticity. It is also well suited for coincidence detection because of its open channel block by Mg^2+^ that must be simultaneously removed in conjunction with its activation by the endogenous synaptic ligands, glutamate and glycine. In addition, the NMDAR plays an important role in intra-neural network activity, where propagating and feedback circuits also can undergo memory related plasticity (Nakazawa et al., 2002; Johnson et al., 2008, 2009; Prager and Johnson, 2009).

Starting with the original study by Paoletti and Ascher (1994), an increasing body of work indicates that NMDAR exhibits mechanosensitivity comparable to inherently mechanosensitive ion channels (Cox et al., 2019). Both prokaryotic and eukaryotic cells have been shown to have mechanosensitive (MS) channels, which play roles in physical signal transduction. In prokaryotes, mechanical signal transduction can be mediated through the force-from-lipids mechanism by protein-lipid interactions directly at the membrane bilayer, whereas in eukaryotes, both “force from lipids” and “force from filament” transduced by protein tethering to the cytoskeleton has been demonstrated (Khairallah et al., 2012; Martinac, 2014; Cox et al., 2019). A growing body of evidence indicates that, in addition to voltage, membrane tension and/or curvature can also remove Mg^2+^ channel block allowing for activation of NMDA receptor channels (Kloda et al., 2007a, b; Parnas et al., 2009; Maneshi et al., 2017). Moreover, mechanosensitive ion channels expressed in neocortical and hippocampal neurons have been shown to exert surprisingly powerful influences on neuronal activities (Nikolaev et al., 2015). This growing evidence invites further exploration of the brain as a “mechanically sensitive organ” and the role mechanical forces play in its function.

## NMDAR in Memory

N-methyl-D-aspartate receptor is essential for normal memory formation. Blockade of NMDAR leads to memory impairment and pathologies which is increasingly being linked to a range of memory related neurological disorders (Newcomer et al., 2000; Morris et al., 2013). NMDAR antagonists have been shown to reduce different types of memories including hippocampal dependent spatial memories and amygdala dependent conditioned memories (Fanselow and Kim, 1994; Mchugh et al., 1996; Morris, 2013). Importantly, NMDAR antagonists impact the formation of long-term consolidated memories and do not impact the retrieval of already established memories. These effects on memory have been demonstrated in experimental *in vivo* models and *in vitro* LTP models of synaptic plasticity (Morris, 2013).

Seminal pharmacological studies linking NMDAR to memory mechanism investigated hippocampal dependent spatial memory using the water maze. Synthesis of the NMDAR specific antagonist D-2-amino-5-phosphonovalerate (APV) enabled investigations into the essential role of NMDAR in memory formation. In comparison to control animals, APV-treated animals failed to form spatial memories of platform location. Control studies confirmed that APV did not affect visual or other sensory systems and the effects were specific to the formation of memory (Morris, 2013). In addition a genetic study, carried out by Wilson and colleagues (Mchugh et al., 1996), also showed that mice whose NMDAR receptors were knocked out in pyramidal cells of the CA1 subregion of the hippocampus suffered deficits in spatial specificity, resulting in navigational learning impairments. Furthermore, a very large body of work has also investigated the role of NMDAR in associative memories, using classical (or Pavlovian) fear conditioning as series of studies investigated the role of associative memories whereby shocks are paired with a specific context or with a specific sound (Ledoux, 2000). This conditioning process leads to the formation of long-term memories that are NMDAR dependent and blocked by APV (Fanselow and Kim, 1994; Maren, 1999; Ledoux, 2000).

The NMDAR ion channel and the subunits contributing to the functional ion channel (see below for subunit details) have been localized to postsynaptic afferent sites as well as to internal excitatory circuits of the hippocampus and amygdala, by electrophysiological and electron microscopy studies. Recurrent circuits necessary for spatial memory pattern recognition in the hippocampus CA3 regions are mediated by NMDA receptors (Nakazawa et al., 2000). Likewise, circuits necessary for Pavlovian fear memory, thalamo-amygdala circuits, as well as amygdala recurrent circuits are mediated by NMDAR (Radley et al., 2007; Johnson et al., 2008, 2009). The thalamo-amygdala afferents target NMDAR containing GluN1 and GluN2B subunits of the NMDAR (Radley et al., 2007). These data identify the presence of NMDAR and especially the GluN2B subunits in circuits for established memory models. The GluN2B unit has also been shown to be essential for LTP formation and mechanosensitivity of the NMDAR (Foster et al., 2010; Singh et al., 2012).

## NMDAR in Long Term Potentiation (LTP)

Memory mechanisms dependent on NMDAR have also been extensively studied using *in vitro* and *in vivo* models of synaptic plasticity. A leading model for this process is LTP of excitatory currents at potentiated synapses. LTP can be induced by high frequency (100 Hz) stimulation of afferent synapses (Bliss and Lomo, 1973), and lower frequency stimulation combined with direct postsynaptic depolarization can also induce LTP (Weisskopf and Ledoux, 1999). Both approaches suggest that LTP needs depolarization activity in the postsynaptic dendrites or spines, induced by repeated high frequency stimulation or by direct depolarization, in combination with presynaptic activity. These findings suggest a role for excitatory synapses and coincidence detection for the formation of LTP (Bliss and Collingridge, 2013).

Several seminal studies of LTP mechanisms have identified the essential role of NMDAR, influx of Ca^2+^ *via* NMDAR, and the essential role of removal of the Mg^2+^ block from the NMDAR pore, for LTP induction. Collingridge and colleagues identified that LTP induction is dependent upon NMDAR (Collingridge, 1987). This discovery was enabled in part by the synthesis of the NMDAR specific antagonist APV (Bliss and Collingridge, 2013). Injection of the Ca^2+^ chelator EGTA into the post synaptic cell prior to LTP induction blocked the LTP, suggesting an essential role for Ca^2+^ influx in LTP mechanisms (Lynch et al., 1983). Interestingly, the NMDAR was also shown to have a low conductance at rest and needed depolarization for increased conduction. This conduction block was mediated by Mg^2+^ in the channel pore [see (Bliss and Collingridge, 2013)]. These findings show that Mg^2+^ block of the NMDAR channel pore can also be removed by mechanical activation, indicating that mechanosensitivity, next to ligand binding and voltage dependence, is another modality of the NMDAR (Kloda et al., 2007a; Maneshi et al., 2017), which opens new lines of enquiry into LTP induction mechanisms.

The contribution and necessity of NMDAR subunits to LTP and memory has also been identified.

Long-term potentiation studies have focused on the necessity for the GluN2B subunit for LTP to be induced and maintained (Gardoni et al., 2009; Foster et al., 2010). Both pharmacological and subunit gene knockout studies have established the importance of the GluN2B subunit. The essential role of this subunit is supported by studies in memory reconsolidation of Pavlovian fear memories, where it was identified within lateral amygdala to be necessary and associated with memory reconsolidation (Nader, 2015). The importance of the GluN2B subunit to memory and its role in the mechanosensitivity of the subunit is further discussed below.

## NMDAR in Memory Related Pathologies

Changes in NMDAR function have been hypothesized to underlie major neurological dysfunctions and psychopathologies. These pathologies potentially include learning and memory deficits, psychosis and excitotoxicity related to cerebral vascular accidence (CVA) and traumatic brain injury (TBI) (Newcomer et al., 2000). Pathological changes in NMDAR may be driven by receptor hypofunction. In age related declines in learning and memory, age has been shown in experimental animal models to be a factor in the decline in NMDAR number and some age-related variations in NMDAR glycine binding sites (see below) (Newcomer et al., 2000). For psychosis, antagonist induced NMDAR hypofunction, driven by subanesthetic doses of ketamine, or more potently, phencyclidine (PCP), can induce a transient psychosis like state (Newcomer et al., 2000). Recently, acute depression has been shown to be responsive to low doses of ketamine suggesting another psychopathology linked to NMDAR hypofunction (Lener et al., 2017a, b). In contrast, hyperfunction of NMDAR, which can be induced by CVA or TBI, can trigger a cascade of excitotoxicity, whereby physical force induced NMDAR activation leads to excessive Ca^2+^ entry triggering neuronal hyperexcitability and cell death, including a cascade of hyperexcitability from the original site of injury to other brain regions (Newcomer et al., 2000). These potential pathologies of NMDAR function highlight the essential need in understanding drivers of NMDAR function including mechanical stimuli, the lipid environment and physical sensation. Thus, all regulators of NMDAR are important for understanding memory associated pathologies including fear, phobias, PTSD, psychoses, and injury.

## Structure of NMDAR

**FIGURE 1:**
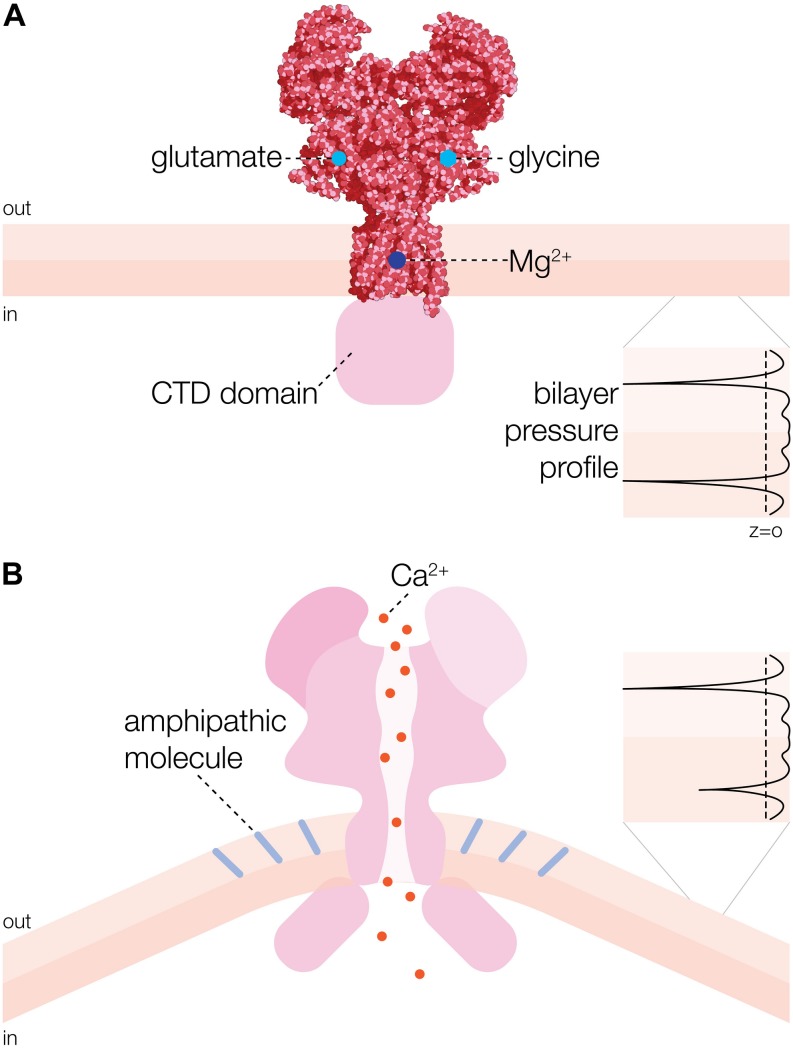
Overall structure and activation mechanism of N-methyl-D-aspartate receptor (NMDAR) ion channel. **(A)** 3D structure of heterotetrameric NMDAR channel consisting of two GluN1a and two GluN2B subunits [adapted from Karakas and Furukawa (2014)]. Glycine and glutamate binding sites are indicated on the GluN1a and GluN2B subunits, respectively. Mg^2+^ binding site is represented by a dark blue circle. CTD refers to cytoplasmic domain. **(B)** Activation of NMDAR channel by insertion of an amphipathic molecule (e.g., arachidonic acid (AA) indicated by light blue rods) into the extracellular leaflet of the membrane bilayer (Casado and Ascher, 1998). One-sided insertion of AA is curving the bilayer, which changes the lateral pressure in the bilayer (black profile inset) causing the channel to open (Bavi O. et al., 2016).

N-methyl-D-aspartate receptor is a heterotetrameric cation channel assembled as dimer of GluN1/GluN2 heterodimers (Lee and Gouaux, 2011; Karakas and Furukawa, 2014) that mediates LTP, synaptic plasticity and neuro-degeneration *via* conditional Ca^2+^ signaling (Marambaud et al., 2009). The structure of the GluN1/GluN2B NMDA receptor ion channel has been solved by X-ray crystallography at 4 Å resolution (Figure 1A; Karakas and Furukawa, 2014). The complexity of NMDA receptors arises from multiple genes encoding different subunits of the channels and alternative splicing of mRNA, which determines the variability in subunit composition, as well as functional heterogeneity (Sugihara et al., 1992; Bradley et al., 2006; Kohr, 2006; Maneshi et al., 2017). Functional channels are comprised of seven known subunits: GluN1, GluN2A-D, and GluN3A-B. The ubiquitously expressed GluN1 subunit is a product of a single gene encoding eight splice variants, whereas four GluN2 subunits (GluN2A–GluN2D) and two GluN3 (GluN3A, GluN3B) subunits are encoded by distinct genes and their products are differentially distributed throughout the central nervous system. The transmembrane topology of NMDAR proteins shows an extracellular N-terminus, followed by three transmembrane domains (TM1–TM3) and intracellular C-terminus. A cytoplasmic re-entrant loop (P) lines the channel pore, whereas an extracellular loop links the TM2 and TM3 domains. In the GluN1 subunit the cytoplasmic C-terminus is the subject of alternative splicing which differentially affects its interaction with intracellular proteins and phospholipids.

## Mechanisms of NMDAR Activation #1: Ligand Binding

Channel activation requires binding of glycine and glutamate to GluN1 and GluN2 subunits, respectively (Hansen et al., 2013), as well as the voltage dependent removal of Mg^2+^ to allow postsynaptic Ca^2+^ influx. This requirement for activation contributes to their role as coincidence detectors and their central role in learning and memory. Importantly, other mechanisms of NMDAR activation and modulation have also been reported, including mechanical stimuli such as membrane tension (Kloda et al., 2007a) and shear force (Maneshi et al., 2017), which is central to this review.

## Mechanisms of NMDAR Activation: #2 Voltage

Voltage-dependent properties of the NMDA receptor that result from a voltage pulse depolarizing the cell membrane combined with the removal of the Mg^2+^-block by depolarization of the cell membrane through the activation of α-amino-3-hydroxy-5-methyl-4-isoxazole-propionic acid (AMPA) receptors, provide a satisfying explanation for the function of this ligand-gated ion channel as a molecular coincidence detector in LTP and memory (Bliss and Collingridge, 1993). Mg^2+^ blocks the NMDA receptor channel from both extracellular and intracellular side with each side having a different Mg^2+^ un-binding rate, suggesting two distinct binding sites, which implies that the NMDA receptor acts as a bidirectional rectifier during synaptic transmission (Johnson and Ascher, 1990). It is the voltage-dependent Mg^2+^-block at the extracellular side of the channel (Zito and Scheuss, 2009), which controls Ca^2+^ permeation and requires removal of Mg^2+^ from a narrow constriction of the selectivity filter by membrane depolarization for LTP and memory formation. This is because high-frequency synaptic transmission in hippocampus, where LTP is mediated *via* NMDAR channels, leads to increased excitation due to summation of AMPA receptor-mediated excitatory post-synaptic potentials. The concomitant reduction in GABA_*A*_ receptor-mediated neuronal inhibition during high-frequency synaptic transmission potentiates further the effect of NMDAR activity. Given that the NMDAR permeability to Ca^2+^ contributes to depolarization of the postsynaptic membrane this leads to further reduction of the Mg^2+^ block and thus to strengthening of LTP and memory function (Bliss and Collingridge, 1993; Bliss and Richter-Levin, 1993).

## Mechanisms of NMDAR Activation #3: Mechanosensitivity

A long-standing and increasing body of work - from different laboratories worldwide - have identified mechanosensitivity properties in NMDAR. In contrast to other ionotropic glutamate receptors NMDAR have been repeatedly reported to have mechanical force regulated gating. Why NMDAR and not AMPA or Kainate receptors have reported mechanical sensing properties is not fully known, here we detail both force from lipids and force from filament mechanisms that may regulate the NMDAR mechanosensitivity. As early as 1992, NMDAR channels have been recognized for potential direct physical interactions impacting on their gating properties (Table 1). By measuring NMDAR current in cerebellar granule neurons, (Miller et al., 1992) identified that arachidonic acid (AA) potentiates these currents. Working in cultured neurons from mouse Paoletti and Ascher (1994) identified that changes in osmolarity and consequent membrane stretch modulated NMDAR currents where reduced osmolarity increased NMDAR currents (Paoletti and Ascher, 1994). Similarly, using cortical and diencephalic cultured neurons, Casado and Ascher (1998) identified that both Lysophospholipids and AA potentiated NMDAR currents (Casado and Ascher, 1998). Working in Dorsal Root Ganglion (DRG) neurons, Chaban et al. (2004) identified that physical poke increased NMDAR dependent Ca^2+^ signals suggesting a mechanical component to the NMDAR mediated Ca^2+^ influx. In 2007 Kloda, Adams, and Martinac using an artificial bilayer preparation found that both membrane stretch and AA could potentiate NMDAR currents (Kloda et al., 2007a). Further studies have identified NMDAR mechanosensitivity properties in a variety of cell types and systems. In 2008, Ma and colleagues, working in renal afferent nerve found that pharmacological modulation of NMDAR and endogenous pressure could modulate urine output. Moreover, they identified the GLuN1 subunit (zeta-1 isoform) in renal nerve (Ma et al., 2008). In 2009, Parnas and Minke, working in both photo receptors and hippocampal CAI neurons found that linoleic acid modulates NMDAR currents and that this lipid modulation could remove the Mg^2+^ open channel block (Parnas et al., 2009).

**TABLE 1:** A brief history of mechanobiology of the N-methyl-D-aspartate receptor (NMDAR).

**Year**	**First author**	**Last author**	**Inst location**	**Journal**	**Cell type/Body region**	**IV**	**DV**	**Summary**
1992	Miller	Atwell	U London	Nature	Cerebellar granule	AA	Currents	AA potentiates currents
1994	Paoletti	Ascher	Ecole N Sup Paris	Neuron	Mouse cultured neurons	Osmolarity and Stretch	Currents	NMDA current increase -reduce osmolarity
1998	Casado	Ascher	Ecole N Sup Paris	J Physiol	Cortical cultures	Lysophosphlipids and AA	Currents	AA potentiates currents
2004	Chaban	McRoberts	UCLA	Neuroscience	DRG	Poke glass pippettes	Ca^2+^ Imaging	Poke increase Ca^2+^ imaging
2007a	Kloda	Martinac	U Queensland	PNAS	Artificial bilayer	Stretch and AA	Currents	AA and Stretch potentiate NMDAR currents
2008	Ma	Lee	Fu Jen Cath U	Hypertension	Renal afferent nerve	Endogenous pressure	Urine output	NMDAzeta1 found in Renal Nerve
2009	Parnas	Minke	Hebrew U	J Neurosci	Photo R and CAI	Linoleic acid (LA)	Currents	Lipid mod removes Open Channel Block
2012	Singh	Meaney	U Penn	J Biol Chem	HEK 293	Stretch	Ca^2+^ Imaging	Stretch sensitivity C term Ser-1323 NR2B
2017	Maneshi	Hua	SUNY Buffalo	Sci Rep	Astrocytes rat	Shear stress	Ca^2+^ Imaging	Shear Stress - blocked by MK801

*Chronology of publications reporting activation of NMDA receptor channels by physical and chemical stimuli that supported the notion of modulation of inherent mechanosensitivity of the channel and modulation of its activity by force from lipids (Note Miller et al., 1992, is included in table as the data has been retrospectively interpreted as relating to force from lipids mechanosensitivity). IV (independent variable), DV (dependent variable).*

Recent studies have not only confirmed the mechanosensitivity of NMDAR but also identified key subunits in the receptor associated with the mechano-properties. In 2012, Meaney and colleagues, working in HEK293 cells identified that physical stretch increased Ca^2+^ entry measured with imaging. Moreover, this group also found that the stretch sensitivity is mediated by the GluN2B subunit and that the C-terminal sequence at Ser-1323 was critical for NMDAR mechanosensitivity (Singh et al., 2012). More recently, Maneshi et al. (2017) working in rat astrocytes found that shear stress increased Ca^2+^ entry and that this effect is NMDAR dependent (Maneshi et al., 2017). Collectively, all these studies provide a substantial and increasing body of work, in different systems and with force applied using different approaches, that collectively identify mechanosensitivity of NMDAR.

Other studies have identified functionally important interactions between cytoskeleton associated proteins and NMDAR that appear necessary for normal NMDAR function and gating and may also be necessary for mechanosensitivity. For example, Rosenmund and Westbrook (1993) identified that actin filaments can directly regulate NMDAR activity. Moreover, calcium and ATP activity can feedback to alter NMDAR activity by directly regulating actin polymerization. Further studies, including immunoprecipitation studies by Sheng and colleagues (Wyszynski et al., 1997) of NMDAR linkages to the postsynaptic cytoskeleton, indicate strong interactions between PSD95 and α-Actinin-2. Importantly, they identified that α-Actinin-2 binds to the cytoplasmic tail of NMDAR subunits including GluN1 and GluN2B (Wyszynski et al., 1997). This is of note as the GluN2B subunit was identified by Singh et al. (2012) as being necessary for NMDAR mechanosensitivity. However, these data suggest actin tethering may be an important mechanism in mechanosensitive properties of NMDAR. NMDAR protein tethering and force from lipids mechanism are not necessarily mutually exclusive, as cytoskeletal tension can interact with bilayer tension (Cox et al., 2016), as shown for Piezo1 channels (Cox et al., 2019). This suggests that, similar to Piezo1 channels, NMDAR mechanosensitivity is driven by a combination of force from lipids and force from filament *via* cytoskeletal interactions with the NMDAR cytoplasmic domain.

The central question of the degree to which NMDAR depends on force from filament *via* cytoskeletal interactions remains unknown as different studies have reported apparently contradictory findings. For example, a classic study by Casado and Ascher (1998) found that GluN1 and GluN2 C-terminus deletions did not affect mechanosensitivity using out-side out and cell attached patch clamp recordings. In contrast in an influential study of NMDAR mechanosensitivity, Meaney and colleagues (Singh et al., 2012) found that mechanosensitivity is governed by the GluN2B subunit. Specifically, they identified a PKC phosphorylation site, Ser-1323 on GluN2B, as a regulator of mechanosensitivity as measured by Ca^2+^ imaging. Explanations for these differences in findings could be dependent on the critical locus of the point mutation on the GluNR2 subunit in confirming mechanical sensitivity and regulating the extent of mechanical sensitivity.

Traumatic brain injury is linked to an increase in intracellular Ca^2+^ leading to excitotoxicity that can cause cell death in both neuronal and glial cells and NMDAR channels have been implicated in this process as well (Hardingham and Bading, 2010). Interestingly, it has been reported that the voltage-dependent Mg^2+^ block of NMDAR current was reduced in mechanically injured neurons (Zhang et al., 1996) suggesting that mechanosensitivity of NMDARs may play a role in TBI. Indeed, a recent study showed that NMDAR channels were mechanically responsive to fluid shear stress in the absence of agonists (Maneshi et al., 2017), which seems also to indicate a physiological role for NMDAR in the glymphatic clearance pathway in the brain (Jessen and Kotz, 2015; Cox et al., 2019; Martinac and Bilston, 2019). Moreover, like mechanosensitive MscS, MscL, TREK-1, TREK-2, TRAAK, Piezo1, and OSCA channels (Kloda et al., 2007a; Brohawn et al., 2014; Cox et al., 2016; Syeda et al., 2016; Murthy et al., 2018), the NMDAR channel protein could be reconstituted into liposomes and activated by stretching liposome patches (Kloda et al., 2007a), which indicates that NMDAR is inherently mechanosensitive meaning that lipid-protein interactions are sufficient for the channel activation by mechanical force.

## Importance of Membrane Lipids for NMDAR Mechanosensitivity

The mammalian brain consists of about 60% fat with fatty acids constituting the major components (Chang et al., 2009). They control many processes essential for the physiology of neuronal and glial cells, including protein trafficking, recognition between cells and production of large numbers of signaling molecules trafficking within and across cells. Long-standing data has shown the potentiating effects of AA on NMDAR conductance through increases in channel open probability. This effect has been suspected to be mediated by modifications to the NMDAR lipid environment. Indeed, released from membrane phospholipids of neuronal and glial cells through the action of cellular phospholipases fatty acids such as AA, phosphatidic acid (PA) and decosahexanoic acid (DHA), play an important role in regulating and modulating activity of membrane receptors, including ion channels. Moreover, the LTP increase in synaptic transmission could be prevented by inhibition of phospholipase A2 (PLA2), since PLA2 hydrolyzes the sn-2 acyl bond of phospholipids causing release of AA (Okada and Miyamoto, 1989) and AA was shown to enhance NMDA receptor-mediated Ca^2+^ entry into hippocampal neurons (Casado and Ascher, 1998; Richards et al., 2003; Table 1). Given that AA is a bioactive amphipathic molecule exerting a direct effect on the membrane lipid bilayer, this result indicates that NMDA receptor channel is able to detect changes in biophysical properties of the membrane bilayer, similar to inherently mechanosensitive bacterial channels MscS and MscL (Martinac et al., 1990; Nomura et al., 2012), which comply with the force-from-lipids paradigm of MS channel gating (Figure 1B; Martinac et al., 1990; Teng et al., 2015). Importantly, insertion of fatty acids into membrane phospholipids can influence membrane fluidity, which has also been shown to affect activity of both MscL and MscS (Ridone et al., 2018) as well as Piezo1 ion channels (Ridone et al., 2019; Romero et al., 2019). Together, these results have firmly established NMDAR as an inherently mechanosensitive ion channel, whose activity in addition to glutamate/glycine, Mg^2+^ and voltage can be modulated directly by mechanical force. More recent data has also implicated intracellular C-terminus of the GluN2B subunit of NMDAR to be essential for its mechanosensitivity (Dela Cruz et al., 2012; Singh et al., 2012). PKC activity on this subunit also appears to be important for mechanochemistry in NMDAR (Singh et al., 2012; Bu et al., 2015).

In addition to fatty acids, lateral membrane heterogeneity in the X-Y direction in the form of the highly ordered and dynamic “raft domains”, also referred to as lipid microdomains, existing on the nanoscale alongside less organized and more fluid regions of the cell membrane (Nicolson, 2014), can modulate the function of NMDAR ion channels (Schrattenholz and Soskic, 2006). These lipid microdomains [which are enriched with cholesterol, sphingolipids and heparan sulfate proteoglycans (HSPG)] have been found to play a critical role for maintenance of synaptic transmission (Hering et al., 2003). Their effect on the function of NMDAR ion channels results most likely from their distinct lipid composition, which is directly linked to biophysical properties of the lipid bilayer (Cox et al., 2019). Consequently, by modulating NMDAR activity lipid microdomains apparently promote LTP and synaptogenesis (Washbourne et al., 2004). Given that amyloid-beta, a neurotoxic peptide characteristic of Alzheimer’s disease, binds to HSPG, it may affect the fluidity of neuronal membrane (Verdier and Penke, 2004; Verdier et al., 2004) and thus cause over-activation of NMDA receptors leading to neuronal excitotoxicity. Also, cholesterol plays an important role in NMDA-dependent Ca^2+^ currents in hippocampal neurons and cerebellar granule cells since its depletion was shown to have a dramatic effect on neuronal excitability (Frank et al., 2004; Korinek et al., 2015), which strikingly resembles the effect of cholesterol depletion on the activity of Piezo1 channels (Cox et al., 2019; Ridone et al., 2019). This is because the cholesterol enriched lipid domains act as “stiffened platforms” for the efficient transmission of mechanical force (Anishkin and Kung, 2013). Since cholesterol is known to affect the bilayer thickness, stiffness, and lateral pressure profile, which have been shown to have a significant effect on the gating of bacterial MscL and MscS channels (Nomura et al., 2012), it may also similarly affect the activation of NMDAR. Nevertheless, the exact mechanism of how lipid microdomains and cholesterol affect NMDAR function and influence neuronal excitability remains unclear and requires further studies of the role lipid-protein interactions play for NMDAR mechanosensitivity.

## Membrane Curvature as a Possible Mechanism of NMDAR Activation by Force-From-Lipids

Curvature can be generated by asymmetric incorporation of amphipathic compounds into lipid bilayers, which depending on the structure of a membrane protein such as an ion channel, can either activate or inhibit the protein function (Cox et al., 2019). The effect of curvature generation on the activity of MS ion channels has been indicated experimentally (Martinac et al., 1990; Perozo et al., 2002; Nomura et al., 2012; Mukherjee et al., 2014; Maneshi et al., 2017) as well as computationally and theoretically (Meyer et al., 2006; Yoo and Cui, 2009; Bavi O. et al., 2016; Kheyfets et al., 2016). Importantly, only local bilayer curvatures of ≤50 nm in radius comparable to a size of an average MS channel oligomer, can generate a change of ∼5–10 mN/m in the transbilayer pressure profile along the membrane bilayer thickness (Bavi O. et al., 2016; Ridone et al., 2018) sufficient to affect the activity of MS channels (Bavi N. et al., 2017), including NMDAR (Kloda et al., 2007a). It is important to note here that the transbilayer pressure profile is an intrinsic property of the membrane lipid bilayer characterized by large stress heterogeneity across the bilayer thickness (Cantor, 1999). This stress heterogeneity is affected by a local bilayer curvature of such dimensions, which is formed in the cell membrane upon activation of phospholipase C (PLC) that cleaves PIP2 in the inner bilayer leaflet resulting in generation of IP_3_ and diacylglycerol (DAG). This is because upon removal of the large inositol phosphate head by PLC a small molecule of DAG is left in the inner leaflet of the bilayer that causes the bilayer to curve locally. The change in the local membrane curvature has been proposed to induce membrane stress large enough to open TRPC6 ion channels, for example (Spassova et al., 2006; Nikolaev et al., 2019). Similarly, the addition of lysophosphatidylcholine (LPC) is thought to generate a local membrane curvature sufficient to activate different MS channels, including MscL, MscS, TREK-1, TREK-2, and TRAAK (Patel and Honoré, 2001; Patel et al., 2001; Nomura et al., 2012). Curving of the lipid bilayer by LPC was demonstrated computationally by molecular dynamics simulations (Yoo and Cui, 2009). The idea that local membrane curvature can be a relevant stimulus affecting MS channel activity is supported by recent determination of the 3D structure of Piezo1 by cryo-electronmicroscopy (cryo-EM) (Guo and Mackinnon, 2017). The cryo-EM structure shows that the shape of the Piezo1 trimer is curving the membrane locally into a dome. When the local dome-shaped deformation becomes flatter upon stretching cell membrane, Piezo1 channel opens by becoming co-planar with the membrane. Together, all the present experimental, theoretical and computational evidence supports the view that curvature is a way to amplify local membrane forces (Cox et al., 2019) to the extent sufficient for activation of MS channels, including NMDAR.

What is the consequence of these findings for the mechanosensitivity of NMDA receptors? The bonus of these findings is that local curvature on the scale of tens of nanometers can be influenced by a host of proteins, lipid types and unilateral insertion of physiologically active amphipathic molecules, such as arachidonic or lysophosphatidic acid (Figure 1; Zimmerberg and Kozlov, 2006; Kloda et al., 2007b; Frisca et al., 2012; Syeda et al., 2016). Moreover, the list of amphipathic molecules produced and released in physiological settings that could modify NMDAR channel function is potentially very long. In addition to unilateral insertion of such amphipaths into the lipid bilayer NMDAR has been shown to be affected by poly-unsaturated fatty acids (PUFAs) (Calon et al., 2005; Keleshian et al., 2014; Islam et al., 2017), which have also been reported to have significant effect on the transbilayer pressure profile and MS channel activity (Ridone et al., 2018). Consequently, we propose that asymmetric insertion of amphipathic compounds into lipid bilayers made of lipids of different poly-unsaturation can be physiologically relevant for the activity of NMDA receptors. Thus, NMDAR channel activation by membrane stretch may be just an epiphenomenon resulting from the fact that membrane tension also affects the transbilayer pressure profile (Bavi N. et al., 2016), which has been postulated to be the common denominator of any mechanical stimuli acting at the membrane interface *via* cytoskeleton and/or extracellular matrix (Cox et al., 2019). However, it is yet to be conclusively shown how TBI, for example, may perturb the transbilayer pressure profile and thus alter the voltage-dependent Mg^2+^-block and trigger Ca^2+^ influx through NMDAR ion channels to cause neuronal excitotoxicity.

In conclusion, an accumulating body of evidence demonstrates mechanosensing properties of NMDAR; however, more work is needed to fully understand the origin and mechanism of the NMDAR mechanosensitivity and how it influences the key neuronal functions of this receptor channel. Under periods of mechanical force including, spine growth, heart rate, and interstitial cerebral-spinal fluid movement, forces can be applied to cell membrane and through combined force from lipids and force from filament to NMDAR, potentially modify memory acquisition and consolidation, thus impacting on both normal and pathological behavior.

## The Function of Mechanosensitivity in NMDAR

The function of NMDAR mechanosensitivity in neurons, and ultimately its role in brain function and behavior, is an important question to explore and identify. Several functional implications for NMDAR mechanosensitivity have been proposed (Miller et al., 1992; Paoletti and Ascher, 1994; Casado and Ascher, 1998; Chaban et al., 2004; Kloda et al., 2007a, b; Ma et al., 2008; Parnas et al., 2009; Kloda and Martinac, 2012; Singh et al., 2012; Maneshi et al., 2017) and here we propose NMDAR gain control mediated by NMDAR mechanosensitivity. Previous proposed functions of NMDAR mechanosensitivity include: (i) amplification of glutamate signaling *via* phospholipase A2 induced AA release was proposed in the early studies of Atwell and colleagues (Miller et al., 1992); (ii) modulation of NMDAR activity in dynamic dendritic spines and growth cones (Paoletti and Ascher, 1994); (iii) NMDAR mechanosensitivity may play a role in TBI when there is clear mechanical force applied to brain (Singh et al., 2012). Additionally, proposed is the role of mechanical modulation of open channel block in NMDAR as an alternative or adjunct to voltage modulation (Kloda et al., 2007a; Parnas et al., 2009). More recently, mechanical force induced during brain development and also the force through ongoing perfusion in the glymphatic system has been proposed as an endogenous modulation of NMDAR mechanosensitivity, where periods of force may interact with the mechanosensitivity of NMDA receptors (Maneshi et al., 2017).

The micro-anatomical structure of neurons can undergo regular physical transformation. Micro- structural plasticity is proposed as a major physical representation of memory (Blumenfeld-Katzir et al., 2011). During memory formation, dendritic spines containing NMDA and AMPA receptors on dendritic spine heads, likely undergo structural modification or enlargement. In addition, structural plasticity underlying memory formation can also include formation of new spines which form synaptic contacts with passing of changing axons (Paoletti and Ascher, 1994; Lendvai et al., 2000; Komiyama et al., 2010; Ozcan, 2017). The formation of new spines is driven by the movement of philopodia, when these membranous structures can protrude from the dendrite on a timescale from seconds and minutes, whereby they may develop into NMDAR and AMPA containing dendrites contributing to the encoding of a memory and also its ongoing stabilization and updating. Furthermore, as highlighted by Paoletti and Ascher (1994), growth cones undergo considerable membrane tension during extension and retraction. NMDAR are also present in these growth cones and direct tension on the membrane in the growth cones will regulate Ca^2+^ entry and axon formation (Paoletti and Ascher, 1994). These plastic, and at times rapid changes to the small micro-structure of neurons, will induce regular membrane shape transformations, which are now known to have large regulatory effects on the NMDAR opening probabilities. Thus, structural plasticity of neurons will induce modification and plasticity to the behavior of NMDAR themselves, which will in turn drive additional plasticity mechanisms.

If NMDAR mechanosensitivity leads to modulation of behavior, a key candidate mechanism is modulation of memory systems in the brain. Moreover, pathology in these mechanisms may lead to altered memory functions as is observed in memory related disorders including PTSD, dementias and TBI. NMDAR mechanosensitivity may be related to memory function through several mechanisms. Early studies by Atwell and colleagues (Miller et al., 1992; Kovalchuk et al., 1994) suggested changes in lipid environment could change thresholds for LTP. Thus, one functional possibility is that mechanosensitivity may serve as a gain control mechanism, whereby increase in AA or other lipid changes exert a continuous force from lipids (Cox et al., 2019) during memory formation that alter thresholds for LTP and memory induction. Further critical studies are needed to investigate the intricate relationship between force from lipids and memory formation.

## Author Contributions

LJ and BM planned and wrote the manuscript. AB prepared the figure. All authors edited the manuscript.

## Conflict of Interest

The authors declare that the research was conducted in the absence of any commercial or financial relationships that could be construed as a potential conflict of interest.
